# Functional DNA Repair Signature of Cancer Cell Lines Exposed to a Set of Cytotoxic Anticancer Drugs Using a Multiplexed Enzymatic Repair Assay on Biochip

**DOI:** 10.1371/journal.pone.0051754

**Published:** 2012-12-31

**Authors:** Anne Forestier, Fanny Sarrazy, Sylvain Caillat, Yves Vandenbrouck, Sylvie Sauvaigo

**Affiliations:** 1 Laboratoire Lésions des Acides Nucléiques, CEA, DSM, INAC, SCIB, UMR-E3 CEA/UJF-Grenoble 1, Grenoble, France; 2 Laboratoire Biologie à Grande Echelle, CEA, DSV, IRTSV, U1038 CEA/INSERM/UJF-Grenoble 1, Grenoble, France; King Faisal Specialist Hospital & Research Center, Saudi Arabia

## Abstract

The development of resistances to conventional anticancer drugs compromises the efficacy of cancer treatments. In the case of DNA-targeting chemotherapeutic agents, cancer cells may display tolerance to the drug-induced DNA lesions and/or enhanced DNA repair. However, the role of DNA damage response (DDR) and DNA repair in this chemoresistance has yet to be defined. To provide insights in this challenging area, we analyzed the DNA repair signature of 7 cancer cell lines treated by 5 cytotoxic drugs using a recently developed multiplexed functional DNA repair assay. This comprehensive approach considered the complexity and redundancy of the different DNA repair pathways. Data was analyzed using clustering methods and statistical tests. This DNA repair profiling method defined relevant groups based on similarities between different drugs, thus providing information relating to their dominant mechanism of action at the DNA level. Similarly, similarities between different cell lines presumably identified identical functional DDR despite a high level of genetic heterogeneity between cell lines. Our strategy has shed new light on the contribution of specific repair sub-pathways to drug-induced cytotoxicity. Although further molecular characterisations are needed to fully unravel the mechanisms underlying our findings, our approach proved to be very promising to interrogate the complexity of the DNA repair response. Indeed, it could be used to predict the efficacy of a given drug and the chemosensitivity of individual patients, and thus to choose the right treatment for individualised cancer care.

## Introduction

Despite active research and the development of target-specific therapies, resistance to standard cytotoxic drugs still represents a challenge in cancer treatment. Many conventional anticancer drugs such as alkylating agents, antimetabolites and topoisomerase inhibitors induce DNA lesions as part of their cytotoxic effect. An important factor that affects the cytotoxic effect of these drugs is the ability of tumour cells to sense a variety of DNA lesions and elicit a coordinated response including activation of transcription, cell cycle arrest, apoptosis and DNA repair processes [1], [2]. This global DNA damage response (DDR) may lead to tolerance to the drug-induced DNA lesions or to enhanced repair [3], [4], preventing an ideal outcome for patients after chemotherapy. The critical importance of the DDR is demonstrated by the existence of mutations in the p53, K-RAS, PIK3CA pathways associated with resistance to treatment [5], [6]. DNA repair mechanisms are a key component of the DDR, representing a set of highly organized pathways which have developed to cope with various types of DNA damage [7]–[9]. Repair of base/sugar modifications – except for strand breaks – is based on excision/synthesis mechanisms. Base excision repair (BER) can deal with small damaged bases and abasic sites [10], whereas nucleotide excision repair (NER) handles helix-distorting lesions [11]. Recently, nucleotide incision repair (NIR) has been described as an alternative to both BER and NER [12]. Some proteins possess overlapping functions within and between BER and NER pathways [13] and proteins ascribed to one pathway can interact with proteins of the other pathways [14]–[16]. Finally, interstrand cross-links (ICLs) are repaired through multiple mechanisms, either recombination-dependent or recombination-independent, with possible cooperation of proteins from NER and mismatch repair (MMR) pathways [17], [18].

Proteins belonging to these DNA repair pathways and to the DNA damage signalling/transducers classes have been identified as potential therapeutic targets [19], [20]. Tumour-specific defects in DDR factors, such as BRCA1/BRCA2, p53, ATM, are now exploited to develop novel specific therapies [19]. Considering the role of the DDR and the various DNA repair pathways in resistance, a better understanding of the mechanisms triggered by direct or indirect DNA-targeting chemotherapeutic drugs is important as it will help to predict the efficacy of the drugs as well as chemosensitivity of individual patients.

Cell lines derived from human tumours represent experimental models of cancers that allow determinants of chemosensitivity to be investigated [21], [22]. Recently, gene expression profiling and other array-based approaches identified specific patterns associated with chemotherapeutic sensitivity [23], [24]. These global strategies also revealed some aspects of the mechanisms of drug action [25]. Sub-classifying cancers according to these new large-scale data is now a strongly emerging concept that raises hope to find more appropriate drugs for tailored treatments [26].

A major limitation that has impeded the understanding of the role of DNA repair mechanisms is the complexity of the DNA repair pathways. Up to now, attempts to determine the role of DNA repair investigated one repair protein at a time [27], [28] which remains of limited power. It is now evident, as stated by Sander and Van Houten [29], that DNA repair must enter into the network biology and protein interactome age. Indeed, the repair response is regulated through adaptive and coordinated mechanisms including protein post-translational modifications and translocations [30], [31]. Consequently, a comprehensive functional approach seems more appropriate than transcriptomic or genomic approaches for the analysis of effective DNA repair efficiency in response to chemotherapeutic drugs.

In the present study, we used a specific multiplexed enzymatic DNA repair assay on biochip to simultaneously investigate several repair pathways. The relevance and advantages of this concept have been demonstrated in aging studies and in an investigation of the consequences of sun photoexposure using human skin cell samples [32]–[34]. We assessed the DNA repair signatures of a panel of 7 cancer cell lines derived from 4 tumour types, treated by 5 cytotoxic anticancer drugs. In particular excision/synthesis repair activities belonging to BER, NER, NIR, and partly ICLs repair were quantified. A rigorous strategy allowed us to present a new effective way of classifying the model cancer cell responses to the chemotherapeutic drugs. It provided new indications on the mechanism of action of cytotoxic drugs, on the ability of cell lines to respond and on the possible involvement of specific repair activities in chemoresistance. Our original approach is an interesting functional complement to molecular pharmacology strategies to understand the DDR and might prove highly effective as part of choosing the right treatment for optimized care of cancer patients.

## Materials and Methods

### Cell culture

Seven cancer cell lines representative of different cancer sites were selected and provided by Oncodesign (France). HCC-1937 and MCF-7 (breast cancer cell line), OVCAR-8/ADR (ovarian cancer cell line, initially misidentified as MCF-7/ADR [35] (Oncodesign)), HCT-15 and HCT-116 (colon cancer cell lines), RPMI 8226 (myeloma), T24 (bladder cancer cell line). All cell lines were cultured in RPMI-1640 containing 2 mM L-glutamine and supplemented with 10% foetal calf serum (Lonza) at 37°C in a CO_2_ incubator (5%). Cell culture, cell treatments and toxicity studies were performed by Oncodesign.

### Chemosensitivity assay

Sensitivity of cell lines to 5 anticancer drugs (cisplatin (CDDP (Sigma), oxaliplatin (OHP (Eloxatine®, DebioPharm)), adriamycin (ADR (doxorubicin, Sanofi Aventis)), 5-fluoro-uracile (5-FU (Sigma)), and carmustine (BCNU (Sigma)) was assessed by MTS (3-(4,5-dimethylthiazol-2-yl)-5-(3-carboxymethoxy phenyl)-2-(4-sulfophenyl)-2H-tetrazolium), Promega) assay. The absorbance was measured at 490 nm using a Victor 3™1420 multi-labelled counter (Wallac, PerkinElmer) (See Methods S1).

The mechanisms of action of the drugs are indicated as Supporting Information, Table S1. The drug concentration leading to 20% mortality was calculated (IC20) and is provided in .

### Treatments and preparation of cell nuclear extracts

Each cell line, plated in a 75 cm^2^ flask (Nunc), was treated at the IC20 with each of the 5 test substances. A control flask for each cell line was also prepared in parallel. After 48 h of treatments, cells were trypsinised, counted and pelleted by centrifugation at 550 g for 10 min. The pellets were suspended in RPMI-1640 medium completed with 10% FCS and 10% DMSO and frozen at −80°C until the preparation of the cell extracts. About 3 10^6^ cells were available per pellet.

Cell nuclear extracts were prepared as already described [32], [36]. Typical protein content was 1 mg/mL.

### Modified plasmid microarray

The modified plasmid microarray has been described elsewhere [33], [36] and is presented as Supporting Information (Methods S1).

### DNA excision/synthesis reaction

Excision/synthesis reaction was conducted on the modified plasmid arrays as described in [33] at a final protein concentration of 0.2 mg/mL for all samples (See Methods S1 for experimental conditions). Each slide comprised 12 identical modified plasmid arrays: 9 arrays were used for the repair reactions performed with the cancer cell line extracts and 3 for control reactions performed with standard commercial HeLa extracts (HeLa_Com; CilBiotech). Each cancer cell line extract was tested in duplicate (technical replicates).

Two independent sets of repair reactions (called Set_1 and Set_2) were performed on cell pellets obtained independently and prepared several months apart. Thus the analysis was performed on data obtained from two independent studies (two experimental replicates).

### Microarray scanning, fluorescence quantification, data treatment and normalization

Images were acquired at 635 nm wavelength at 10 µm resolution using a Genepix 4200A scanner (Axon Instrument). Total spot fluorescence intensity (FI) was determined using the Genepix Pro 5.1 software (Axon Instrument). Within each set of reactions, duplicate data collected from the cancer cell line experiments were normalized using NormalizeIt software [36]. Then, for each test sample, we determined an intensity value for the 6 modified plasmids corresponding to the sum of the intensities of the A, B and C dilutions from which the value for the CTRL was subtracted. This value corresponded to the intensity of the unmodified Control plasmid multiplied by 3, for consistency with regard to the sum of 3 plasmid dilution intensities. Therefore, each sample was characterised by 6 values, corresponding to the repair of the 6 DNA lesions represented on the biochip.

As the two separate experiments (Set_1 and Set_2) were conducted on different plasmid microarray batches on different days, we had to correct for inter set and inter day fluorescence variations attributed partly to changes in the ozone level [37]. The strategy used for data treatment and normalization is provided as Supporting Information (Experimental Work Flow Fig. S1).

### Data expression

Effect of treatment on the different enzymatic DNA repair activities was examined through the calculation of the ratio of FI obtained for each lesion and each treatment condition, between treated (T) and non-treated (NT) samples. Ratios were transformed to log2, so that stimulated and inhibited repair with respect to non-treated cells was centred on zero and exhibited values of opposite sign. These values are reported as log2(T/NT). Note that in Set_2, 4 treated cell lines (RPMI8226_5-FU, HCT-116_ADR, HCT-116_BCNU and MCF7_OHP) presented repair intensities not significantly above background. The corresponding log2 ratios were set to the minimum of the whole data set (minus 1).

### Data analysis - Clustering methods - Results display

Unsupervised hierarchical clustering was used to explore the structure of the dataset, to describe and visualise the relationship between the different treatments and the different cell lines. The analysis was performed using the free software environment for statistical computing and graphics, R (http://r-project.org/). The hierarchical average linkage clustering algorithm was run with two different dissimilarity measures (1) the Euclidean distance, which aggregates profiles with both similar intensity levels and covariation, (2) the correlation dissimilarity measure (1 – r), where r indicates the Pearson correlation coefficient, which aggregates profiles with similar covariation independently of their intensity levels. Thus two complementary classifications were obtained that explore the data differently, one considering both co-regulation of repair pathways and intensity level and the other considering only co-regulation of repair pathways regardless of the intensity level (See Methods S1).

### Investigation of the relationship between DNA Repair Response (DNA-Rep-Res) and chemosensitivity

To explore the association between the IC20 obtained for each drug and the DNA-Rep-Res, we performed unsupervised hierarchical clustering using the Euclidean dissimilarity measure and average linkage agglomeration method. IC20 was chosen as, at this mild level of toxicity, cells are expected to be able to induce a specific DDR, providing drug-specific exposure signatures. The mean log2(T/NT) FI of the 2 sets of experiments for each lesion-treatment association was plotted against the log10(IC20) of the corresponding treatment. Values along each axis were standardized within each cell line series (mean = 0 and standard deviation = 1). The optimal number of clusters was determined by cutting the cluster dendrograms at the agglomeration criteria inflexion point (Fig. S2). The partitions of treatment-lesions obtained for each cell line as a function of IC20 could be easily visualised on two-dimensional coloured charts.

### Statistical tests to evaluate degree of similarity

We used the “pvclust” R package to assess the uncertainty in hierarchical cluster analysis (http://www.is.titech.ac.jp/~shimo/prog/pvclust/). Clusters with AU *P* value>95% were considered significant (See Methods S1 for details).

## Results

### Sensitivity of cell lines to anticancer drugs: classification according to IC20 (Fig. 1)

**Figure 1:**
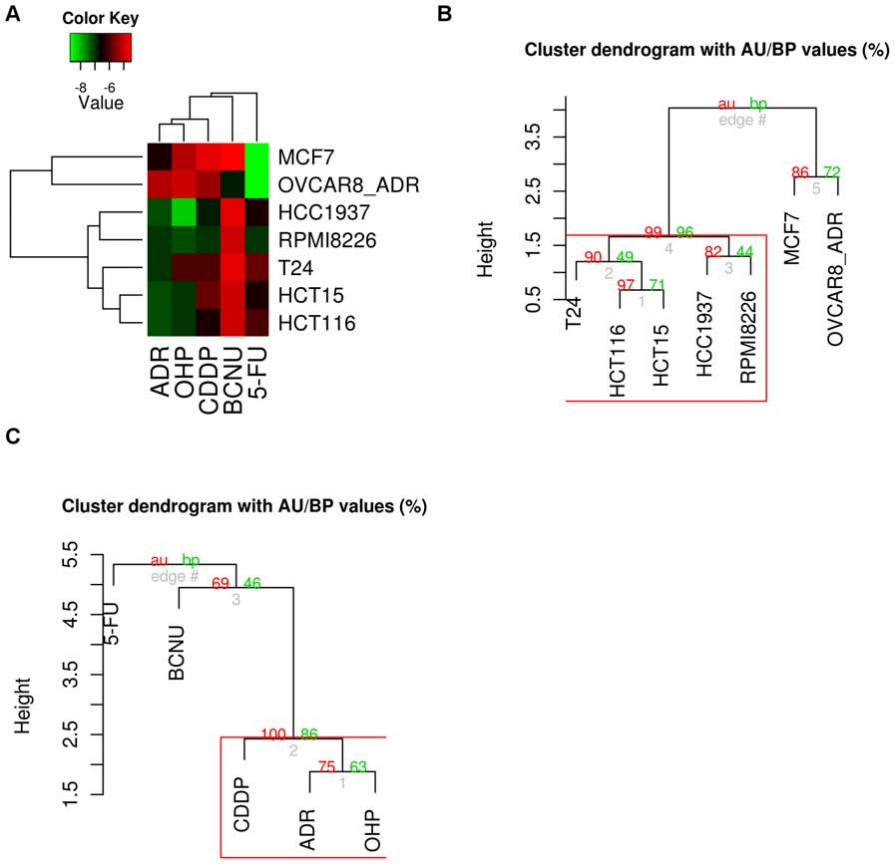
Hierarchical clustering of the cell lines and of the drugs. Based on the log10(IC20), the clustering used the Euclidean dissimilarity measure. A. Heat map representation of the clusters. Brightness of the colour is correlated with log10(IC20), with red colour for higher IC20 and green for lower IC20. B. Cell lines dendrogram with clusters significance values. **C**. Treatments dendrogram with clusters significance values. *P*-values (AU (Approximately Unbiased) in red and BP *P* values (Bootstrap Probability) in green are reported on the dendrograms.

We wished to induce DNA damage without inducing excessive apoptosis or necrosis in the 7 cell lines tested, as apoptotic and necrotic cells contain high levels of nucleases. These nucleases would interfere with our downstream analysis. IC50 is commonly used in pharmaceutical studies to test compounds and their toxicity towards cells, but we preferred to use a milder level of toxicity, IC20, so as to trigger a response from the cells without inducing excessive cell death. The 7 cell lines and the 5 treatments were clustered according to log10(IC20) using the Euclidian dissimilarity measure. In the first dimension, the cell lines were clustered by similarity of their log10(IC20) profile across the 5 treatments. In the second dimension, the treatments were clustered by similarity of their log10(IC20) profile across the 7 cell lines. In the colour-coded grid, cell lines and treatments have been listed in the two dimensions, and each grid block shows the log10(IC20) value for each cell line and treatment. Brightness of colour is correlated with log10(IC20), with red for higher IC20 and green for lower IC20 (Fig. 1A). Cell lines were separated into two clusters represented by the two major dendrogram branches: MCF7 and OVCAR-8/ADR on one side with a very low IC20 for 5-FU and very high for the other treatments (except BCNU for OVCAR-8/ADR and ADR for MCF7). On the other side, the other cell lines were grouped together, with a higher IC20 for BCNU than for the other treatments, all of which had an intermediate IC20. Inside this cluster, HCT-116 was significantly closer to HCT-15, showing a similar sensitivity of the two colon cancer cell lines for the different treatments (Fig. 1B). The treatments could be divided into 3 different groups: on the one hand, CDDP, ADR and OHP were grouped together, with IC20 for MCF7 and OVCAR-8/ADR higher than for the other cell lines. 5-FU was opposed to this group, with IC20 for MCF7 and OVCAR-8/ADR much lower than for the other cell lines. Finally, BCNU was classed separately, with a much higher IC20 for every cell line except OVCAR-8/ADR (Fig. 1C).

### DNA Repair Response profiling: classification of the cell lines according to the effect of treatment on the DNA repair activities (Fig. 2)

We used the complete data set (log2(T/NT) values from Set_1 and Set_2) to cluster the DNA-Rep-Res (represented by the repair of the 6 lesions) and get an overview of similarities across the cell lines and treatments. Importantly, this also allowed us to evaluate the consistency between the two independent sets of experiments.

**Figure 2:**
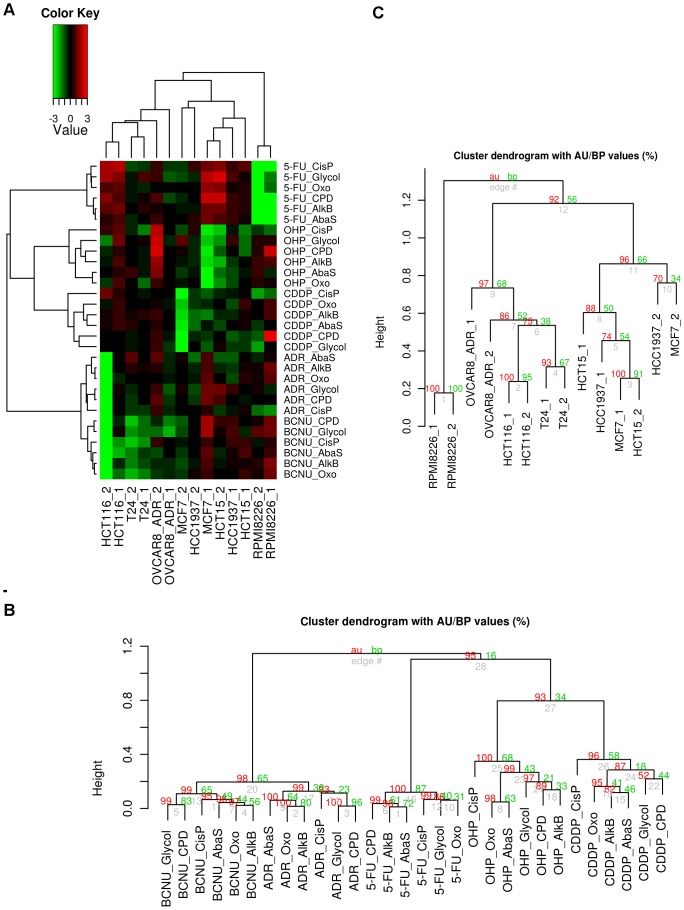
Analysis of the DNA repair response of the cell lines across treatments and lesion types. A. The heatmap was constructed using the log2 transformed ratios of the fluorescence intensity obtained for repair of each lesion between treated and non-treated cell lines (log2(T/NT)). Hierarchical clustering algorithm with correlation dissimilarity measure was used to group in a colour-coded grid the seven cell lines from the two experiments and the five treatments by lesion type repair, in the two dimensions. In the first dimension, cell lines were clustered by similarity of their DNA repair response profile covariation across the impact of the five treatments by lesion type repair. In the second dimension, treatments by lesion type repair were clustered by similarity of their pattern covariation across the seven cell lines from the two experiments. To evaluate the consistency between the two independent experiments, Set_1 and Set_2 data were kept separated and analyzed simultaneously (marked _1 and _2 respectively). In the colour-coded grid, values greater than 0 are shaded in red indicating stimulation of repair activities whereas values below 0 are coloured in green indicating an inhibition of repair activities, compared to NT cells. Values greater than 0 were shaded in red indicating stimulation of repair activity, while values below 0 were shaded in green indicating inhibition of repair activity. Values around 0 were coloured in black indicating no detected effect. Brightness of colour was correlated with the magnitude of effect of treatments on the DNA repair activities. B. Dendrogram of the five treatments by lesion type repair with clusters significance values. C. Dendrogram of the seven cell lines from the two experiments with clusters significance values. P-values (AU (Approximately Unbiased) *P* value in red and BP (Bootstrap Probability) *P* value in green) are reported on the dendrograms.

Clustering with correlation dissimilarity measure was used to group the 7 cell lines from the two experiments and the 5 treatments by lesion type (corresponding to repair pathway), in the two dimensions. In the first dimension, cell lines were clustered by similarity of their DNA-Rep-Res covariation across the 5 treatments using the 6 lesion types. In the second dimension, treatments associated with the 6 lesion types were clustered by similarity of their covariation pattern across the 7 cell lines from the two experiments (Fig. 2A). The heatmap offered a comprehensive overview of the classification where the differences between the treatment-induced phenotypes with respect to the identity of the cell lines could be easily visualised.

The dendrogram of the treatments by lesion types (Fig. 2B) showed that the lesions remained clustered by treatment type, indicating that each drug had a distinct effect on all repair pathways simultaneously. This analysis further revealed three classes of drugs: one encompassing BCNU and ADR treatment (AU p-value>95%) and another one encompassing OHP and CDDP treatment (AU p-value>93%). 5-FU was apart, although it tended to cluster with the second group. Moreover, as a general feature, when the 6 lesion types were clustered on the set of cell lines by treatment, between the two sets of experiments it consistently appeared that 8oxoG (8oxoG), alkylated bases (AlkB) and AP sites (AbaS), all repaired by BER, tend to be clustered together; whereas Glycol and photoproducts (CPD-64) grouped independently. Cisplatin (CisP) formed a group alone (Fig. S3). When each treatment was considered separately, this trend was maintained for BCNU and ADR treatment, albeit with some differences (Fig. 2B). On the contrary, notable differences appeared following treatment with 5-FU. Interestingly, in the case of CDDP and OHP treatment, the CisP repair pathway was significantly distinct.

In the cell lines dendrogram, the cell lines were organized in 3 significant main groups (Fig. 2C). A separate branch contained RPMI8226, a myeloma-derived haematopoietic cell line that, unlike all the other cell lines, is not derived from a carcinoma. The two mammary carcinoma cell lines, MCF7 and HCC1937, belonged to the same cluster, together with HCT-15. In this subgroup, the replicates for each cell line were mixed-up. The replicates of the other colon cancer cell line HCT-116 constituted a subgroup of a cluster containing OVCAR-8/ADR and the bladder carcinoma cell line T24.

Importantly, we observed that the two independent replicates for each cell line were mostly closely associated, reflecting the repeatability of the experiment and, consequently, the reliability of the approach. The correlation dissimilarity measure relies only on co-regulation and is independent of any batch effect between experiments. This measure was more robust than the Euclidian distance when demonstrating the close similarity between the replicates.

### Impact of the drug treatments on DNA Repair pathways: stimulation or inhibition

**Figure 3:**
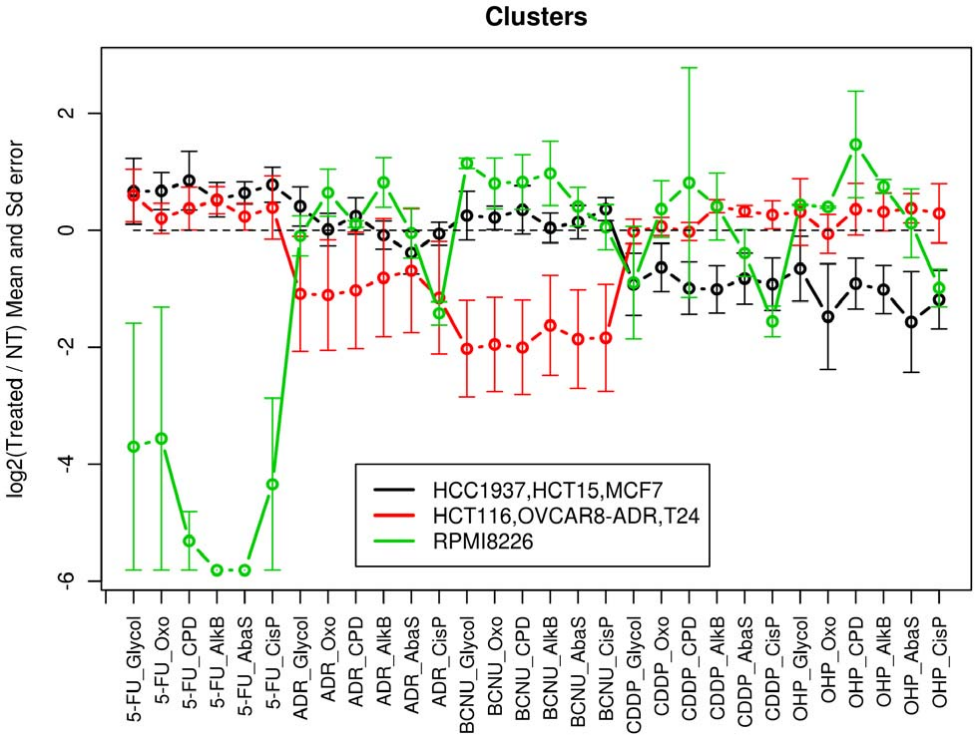
Characteristics of the 3 cell line clusters identified. Means and standard errors of the three identified classes of cell line responses across the five treatments and the six lesion types were calculated using the log2(T/NT) data (black line: HCC1937, HCT-15, MCF7; red line: HCT-116, OVCAR-8/ADR, T24; green line: RPMI8226).

To distinguish classes of responses to treatments with respect to the different DNA repair sub-pathways, the means and standard errors of the data (log2(T/NT) for each repair pathway) within the 3 main cell line clusters identified were represented on the same chart. This allowed easy visualisation of the 3 profiles (Fig. 3). To characterise each cluster, significant stimulation or inhibition of the different repair sub-pathways was subsequently investigated. For this purpose, we applied statistical hypothesis tests to each of the 2 clusters containing 3 cell lines. As the data distribution could be non-Gaussian, the non-parametric Wilcoxon test was used. For each cluster and treatment by lesion type, the Wilcoxon test determined whether the median of log2 ratio distribution was significantly different from 0, highlighting stimulating (if median >0) or inhibiting (if median <0) effects. For the cluster containing only one cell line (RPMI8226), stimulation or inhibition was considered significant when |mean|>3×standard error. Results are displayed in Table 1.

**Table 1:** Characteristics of the 3 cell line clusters in terms of DNA repair sub-pathway response to the drugs treatments (see Fig. 3).

Treatment	CDDP	OHP	ADR	5-FU	BCNU
Cluster					
HCC1937, HCT-15, MCF7	Glycol* CPD-64* AlkB* AbaS* CisP**	8oxoG** CPD-64* AlkB** AbaS** CisP*		8oxoG* AbaS* CisP*	
HCT-116, OVCAR-8/ADR, T24	AlkB*AbaS*		8oxoG*	AlkB*	All lesions*
RPMI-8226	**CisP** (−1.56±0.27)	*Glycol* (0.43±0.01) *8oxoG* (0.40±0.02) *AlkB* (0.75±0.12)	**CisP** (−1.42±0.2)	**CPD-64** (−5.31±0.5) **AlkB** (−5.81±0) **AbaS** (−5.81±0)	*Glycol* (1.14±0.09)
		**CisP** (−0.99±0.32)			

**Text in bold** indicates the repair activities significantly inhibited.

*Text in italics* indicates the repair activities significantly stimulated.

For the two clusters composed of three cell lines, the Wilcoxon test was used to investigate if repair toward the different lesions was either stimulated or inhibited. Results with *P* value<0.05 (noted **) or <0.1 (noted *) are reported. For RPMI-8226 cluster, as only two data by treatment and lesion type were available, repair was considered either stimulated (positive value) or inhibited (negative value) when |mean|>3×standard error. For each cluster and treatment, lesions exhibiting significant repair inhibition after treatment were displayed in bold, and lesions exhibiting significant repair stimulation after treatment were displayed in italics.

Depending on the nature of the drug, the number of affected repair pathways varied. Among features that emerged, we observed that 5-FU drastically significantly inhibited CPD-64, AlkB and AbaS repair in RPMI8226 cell line. CDDP, OHP and ADR all appeared to inhibit the CisP repair pathway of RPMI8226 cell line compared to the others repair pathways investigated. By opposition, with OHP treatment, other repair pathways of RPMI8226 (Glycol, 8oxoG and AlkB) were significantly up-regulated. BCNU exerted an inhibitory effect on all repair activities in the [HCT-116, OVCAR-8/ADR, T24] cluster with *P* value<0.1. Interestingly, ADR inhibited repair of 8oxoG only within this cluster (*P* value<0.1). The two platinum-based anticancer drugs, CDDP and OHP, did not affect repair activities in the [HCT-116, OVCAR-8/ADR, T24] cluster apart from stimulating repair of AlkB and AbaS for CDDP (*P* value<0.1). Some repair activities in the [HCC1937, HCT-15, MCF7] cluster were slightly up-regulated by 5-FU treatment (significant for 8oxoG, AbaS, CisP with *P* value<0.1) and clearly down-regulated by the platinum-based drugs (see Table 1 for significance). Conversely, ADR and BCNU treatments did not have any impact on the DNA repair pathways of this cluster.

### Impact of the drug treatment: analysis of cell line response treatment by treatment

When we focused independently on each treatment, additional peculiarities were highlighted. This concerned, in particular, the two colon cancer cell lines HCT-15 and HCT-116 treated by 5-FU. Both cell lines displayed stimulated DNA repair activities, whatever the pathway considered (One-sided Wilcoxon test; *P* value = 0.065) (Fig. S4A). A new cell line cluster shared similarities when treated by BCNU: HCC1937, RPMI8226, and HCT-15 for which repair of 8oxoG and CPD-64 were stimulated (One-sided Wilcoxon test; *P* value = 0.05) (Fig. S4B).

### Investigation of the relationship between DNA Repair Response and chemosensitivity

**Figure 4:**
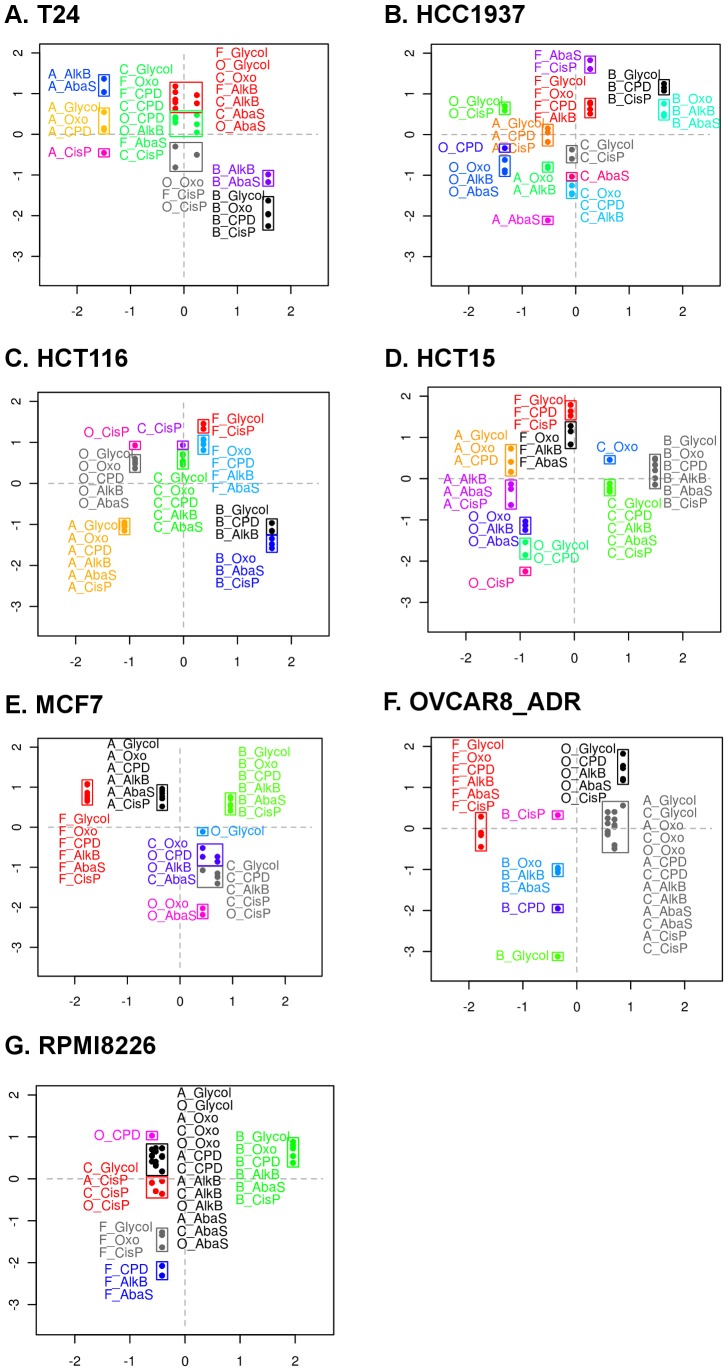
Visualisation of the association between the DNA repair response (sub-pathways) and chemosensitivity. We reported, for each cell line, the means of log2(T/NT) data (Y-axis) gathered for each treatment of the 2 sets of experiments as a function of the corresponding log10(IC20) (X-axis). Data were standardized within each cell line series (mean = 0 and standard deviation = 1). Within each chart, the lesion-treatment associations belonging to the same cluster were framed together. The corresponding cluster dendrograms are displayed in Supplementary Fig. S2 (the letters ahead of the lesions refers to the treatment: A = adriamycin; B = BCNU; C = cisplatin; F = 5-FU; O = oxaliplatin).

The graphical representations of the log2(T/NT) data as a function of log10(IC20) allowed meaningful visualisation of the clusters which represented co-regulated sub-pathways for each drug-induced DNA-Rep-Res (Fig. 4 A–G). For convenient visualisation of the data within each chart, DNA repair sub-pathways belonging to the same cluster were framed together. Depending on the cell line considered, the number of clusters ranged from 6 to 13 classes. Within each graph, repair sub-pathways related to one treatment could be either grouped (e.g. HCT-116, Fig. 4C and MCF7, Fig. 4E) or scattered (e.g. HCC1937, Fig. 4B, HCT-15, Fig. 4D). We can assume that this latter feature revealed distinct regulations of the different repair sub-pathways, in turn responsible for the increase of cluster number.

Considering the dimension of the data, in this paper we could focus only on obvious associations, such as low repair/low IC20 and high repair/high IC20, susceptible to reflect a causality link between repair efficiency and chemosensitivity. This does not exclude that other associations might be biologically relevant.

For example, the HCC1937 chart displayed a lower repair of abasic sites in response to ADR treatment (ADR-AbaS) associated with a lower IC20 compared to other treatments (Fig. 4B). Conversely, a BCNU-driven higher level repair of all lesions was associated with elevated IC20 (Fig. 4B). HCT-116 exhibited an association between the whole ADR-driven repair response and IC20, both being low (Fig. 4C). Another feature worth mentioning concerned HCT-15, where low repair of CisP following OHP treatment was associated with low IC20 (Fig. 4D). In the case of RPMI8226, the high BCNU-driven response was associated with a high IC20 (Fig. 4G).

By opposition, inverted associations were also noted such as for T24 where ADR treatment was the most cytotoxic (low IC20) and nevertheless associated with the highest DNA-Rep-Res (Fig. 4A). Importantly, the fact that ADR DNA-Rep-Res and low ADR-IC20 were inversely associated did not mean that they were formally anti-correlated and no hypothesis could be formulated for the moment regarding the biological meaning of such potential anti-correlation. The same observation was found in the case of MCF7 treated with 5-FU (Fig. 4E). Finally, the two colon cancer cell lines HCT-116 and HCT-15 exhibited a similar response to 5-FU, with rather weak toxicity, but could be discriminated by at least ADR and OHP treatments (Fig. 4C and 4D respectively).

## Discussion

In this study we took advantage of a multiplexed functional approach that quantifies DNA repair activities to explore the DDR and the relationship between drug-induced cytotoxicity and DNA repair sub-pathways. We used statistical approaches and privileged visual representations that clearly displayed our findings. For the first time, to our knowledge, this strategy organized the DNA repair phenotypes on the basis of their patterns and provided new insights into both similarities across cell lines and similarities across drugs.

The six repair sub-pathways investigated here, represented by the six lesions present on the biochip, tended to cluster in three groups. 8OxoG, AlkB and AbaS, all repaired by BER, grouped together whereas CPD-64 and Glycol constituted another group and CisP was apart. We believe that this clustering is a reflection of the dominant repair regulation pathways. What is interesting to stress here is that when the treatments were considered separately, this feature was disrupted. As a matter of fact, different discriminations between the sub-pathways were observed highlighting specific impact of drugs on certain repair sub-pathways according to the lesions they induced.

Within the DNA-Rep-Res classification, cell lines derived from identical tissue sites tended to cluster together. Hence, in Fig. 2, one can note that the myeloma RPMI8226 cell line, the only non-carcinoma cell line, is in a distinct cluster. The two mammary carcinoma cell lines HCC1937 and MCF7 were grouped. An exception concerns the two colorectal carcinoma cell lines that exhibited different DNA-Rep-Res although both are MMR deficient. This is an important indication that other key genes within these cell lines might drive the DNA-Rep-Res.

Clearly, each drug exerted a general specific effect on the cell lines tested, as revealed by the hierarchical classification assigning them to different branches (Fig. 2). However, significant drug subgroups also appeared. In the first group, composed of BCNU and ADR, an alkylating drug is associated with an intercalating agent. Chloroethylating agents like BCNU have complex effects [38]. Even though the O6 position of guanine is an important target for alkylation, BCNU also forms ICLs [39]. ICLs repair requires NER factors as well as proteins from homologous recombination pathways [38]. On the other hand, adriamycin is a DNA intercalator which prevents topoisomerase from binding DNA and blocks DNA relegation at low concentration [40]. In addition, it forms covalent adducts that exhibit characteristics of ICLs [41]. Thus our finding is consistent with a grouping of BCNU and ADR on the basis of their ability to form ICLs, impacting in a similar way the DDR and the DNA repair mechanisms.

Logically the two platinum-based anticancer agents, cisplatin (CDDP) and oxaliplatin (OHP), were found in the same group indicating that globally they trigger similar DNA-Rep-Res. Recognition of the cisplatin adducts modulates several signal transduction pathways involving AKT, p53 and MAPK [42]. This supports a general effect of this drug on the DDR. Another point worth mentioning for the DNA-Rep-Res induced by these 2 drugs concerned the position of the CisP lesion in a completely separate branch of each of the two clusters (Fig 2B). Like for photoproducts, NER is the major pathway involved in the removal of most cisplatin adducts. A difference between these two lesions comes from the recognition of 1,2-intrastrand crosslink platinum-DNA adducts by high-mobility group (HMG) box proteins [42], [43] that could be responsible for the discrimination observed between CPD-64 and CisP repair. Here, the mechanism of action of the drug is reflected by a clear signature at the level of the DNA repair pathway that precisely takes charge of this drug-induced lesion. This feature of CDDP and OHP clusters exemplifies the fact that both global DDR and specific DNA repair mechanisms account for the DNA-Rep-Res observed.

Finally, the only antimetabolite used in this study, 5-FU, was isolated in the clustering. This compound exerts its cytotoxicity via inhibition of thymidilate synthetase and incorporation into RNA and DNA [44]. BER and MMR are the two probable mechanisms in charge of the 5-FU-induced DNA lesions although it appears that Homologous Recombination (HR) could play a compensatory role [45]. These characteristics of 5-FU action resulted in a rather unique signature in the classification.

From the examination of the DNA repair profiles of cell clusters on Fig. 3, we hypothesize that the three different patterns of DNA-Rep-Res probably characterise three different combinations of functional/dysfunctional proteins belonging to cascades of signalling pathways and/or associated effectors. Therefore the DNA-Rep-Res could serve as surrogate endpoint to get comprehensive information on the overall functionality of signalling pathways.

Within each cluster, distinctions between repair sub-pathways were revealed (see Table 1). These differences in terms of stimulation/inhibition deserve further validation and exploration as they could be important biomarkers of drug effects and cell response.

Both colon cancer cell lines are mutated for MMR, although on different genes (MLH1 and MSH6 for HCT-116 and HCT-15, respectively [46]). The DNA-Rep-Res was stimulated by 5-FU treatment (Fig. S4A) whatever the repair pathway considered. This observation suggests that a common upstream mechanism, up-regulated by the treatment, would impact all downstream effector pathways. The S-phase checkpoint signalling pathway is a good candidate to play this role. It has been reported to respond to 5-FU treatment and thymidilate synthetase inhibition by ATM and ATR activation [45] that in turn stimulates the repair pathways through p53 phosphorylation [47]. There is, however, controversy surrounding this, as 5-FU adjuvant treatment is considered beneficial for MMR-defective colon cancer patients [48], [49]. Although both cell lines responded similarly to 5-FU, they differed with respect to their response to the other treatments. This indicates that other driving genes determine the sensitivity of these cells to treatment and might explain the discrepancy observed in the MMR-deficient tumours. Clearly, based on our criteria, they should not be considered as a homogenous group. Consequently, our approach might help identify subsets of colon cancer types that could benefit from distinct combination therapies according to their overall DNA-Rep-Res.

The relationship between the DNA-Rep-Res and cytotoxicity is complex. In particular the contribution of DNA repair capacity to cell resistance to drug could not be directly investigated here. A common hypothesis is that a poor DNA repair capacity is associated with higher cytotoxic effect of DNA-damaging drugs and that defective damage-related signalling pathways may lead to cell death [50]. Conversely, high repair capacity is supposed to result in chemoresistance [51]. To determine the possible link between chemosensitivity and DNA repair, we investigated the association between the IC20 obtained for each drug and specific repair sub-pathways independently for each cell line. We identified associations between up-regulations of certain repair pathways and high IC20, as well as the inverted feature (low repair/low IC20). It is reasonable to assume that if the DNA repair mechanisms are directly responsible for resistance, proportionality will be observed between drug concentration and level of DNA-Rep-Res. Experiments with different drug concentrations should be conducted to study this dose/response relationship. Selective inhibition of the putative DNA repair pathways responsible could also be envisaged to probe the biological mechanisms. Therefore, our experimental strategy raises hypotheses as to the role of DNA repair in chemoresistance, which must then be tested. All drugs used here are genotoxic, DNA-damaging agents. Therefore the specific pattern observed for the DNA-Rep-Res as a function of IC20 illustrates how our strategy could also be used to class genotoxics according to the DDR they trigger and could contribute to elucidating their mechanism of action.

### Concluding remarks

Because DDR and DNA repair mechanisms constitute a dynamic network of finely tuned pathways with coordinated back-up and redundancy, comprehensive functional assays that enable DNA repair activities to be measured are promising. We showed that this hypothesis-generating strategy provided new, novel information on the cell lines and on drugs studied. Specific DNA repair signatures could represent in the future new prognostic and predictive biomarkers of patient response and drug efficacy, thereby potentially leading to the development of more personalized treatments. As an adapted DNA repair response reflects the nature of the DNA lesions the cells have to handle, the DNA repair phenotype of a given exposed cell type can be considered as a marker of the mechanism of action of a drug. The knowledge of the specific DNA repair sub-pathways induced will provide important clues as to the dominant DNA lesions formed that are responsible for the cytotoxic effect. In addition, classification of DNA-Rep-Res obtained using model and in-development compounds can help to decipher mechanisms of action based on similarity of DNA-Rep-Res profiles. Finally, our functional and multiplexed approach could be integrated into systems biology approaches for more effective identification of cancer biomarkers.

## Supporting Information

Figure S1 **Experimental workflow.**
(DOC)

Figure S2 **For each cell line, treatments by lesion type were clustered according to their repair response and IC20 (standardized data).** Unsupervised hierarchical clustering was performed, using Euclidean dissimilarity measure and average linkage agglomeration method. The cluster dendrogram is displayed together with the agglomeration criteria of average linkage method. To get a partition of the data, the resulting cluster dendrograms were cut at the agglomeration step (represented by the dots of the red line) corresponding to the optimal number of clusters indicated by the agglomeration criteria inflexion point. This latter operation determined the number of clusters identified (A: T24, 8 clusters identified; B: HCC1937, 13 clusters identified; C: HCT-116, 9 clusters identified; D: HCT-15, 10 clusters identified; E: MCF7, 7 clusters identified; F: OVCAR-8/ADR, 7 clusters identified; G: RPMI8226, 6 clusters identified).
(TIF)

Figure S3 **Clustering of the DNA repair pathways, represented by the lesions.** The 2 sets of experiments (Set_1 (A) and Set_2 (B), noted _1 and _2) were clustered, using the Euclidian dissimilarity. Similar results were obtained when the correlation dissimilarity was considered. Four treated cell lines presenting unquantifiable repair (very low signals) in Set_2 were removed from the data set since their atypical profiles with very low log2 ratios had an overly strong influence on clustering (RPMI8226_5-FU, HCT-116_ADR, HCT-116_BCNU and MCF7_OHP).
(TIF)

Figure S4 **Analysis of the DNA repair response clustering independently for cells treated with 5-FU (A) and BCNU (B) using the Euclidian dissimilarity.** This analysis provided additional data for Fig. 3. A. RPMI8226 remained apart, whereas the two other cell line clusters previously identified clustered as a single significant group. The 5-FU treatment, in particular, significantly stimulated all repair activities in the two colon cell lines, HCT-116 and HCT-15 (one-sided Wilcoxon test; *P* value = 0.0625). B. A new significant cell line cluster sharing similarities in response to BCNU (stimulation of 8oxoG and CPD-64 repair activities) was identified: [HCC1937, RPMI8226, HCT-15] (one-sided Wilcoxon test; *P* value = 0.05).
(TIF)

Table S1 Mechanism of action of the drugs used.
(DOC)

Table S2 IC20 expressed in molar concentration determined by MTS test.
(DOC)

Methods S1 **Supplementary information on the following: Chemosensitivity assay, modified plasmid microarray, preparation of cell nuclear extracts, DNA excision/synthesis reaction, data normalization, statistical tests to evaluate degree of similarity, data analysis - clustering methods - results display.**
(DOC)
