# Supplementary figures and images for: Functional DNA Repair Signature of Cancer Cell Lines Exposed to a Set of Cytotoxic Anticancer Drugs Using a Multiplexed Enzymatic Repair Assay on Biochip
Source: PLoS One. 2012 Dec 31;7(12):e51754. doi: 10.1371/journal.pone.0051754 (PMC3534104; doi:10.1371/journal.pone.0051754)

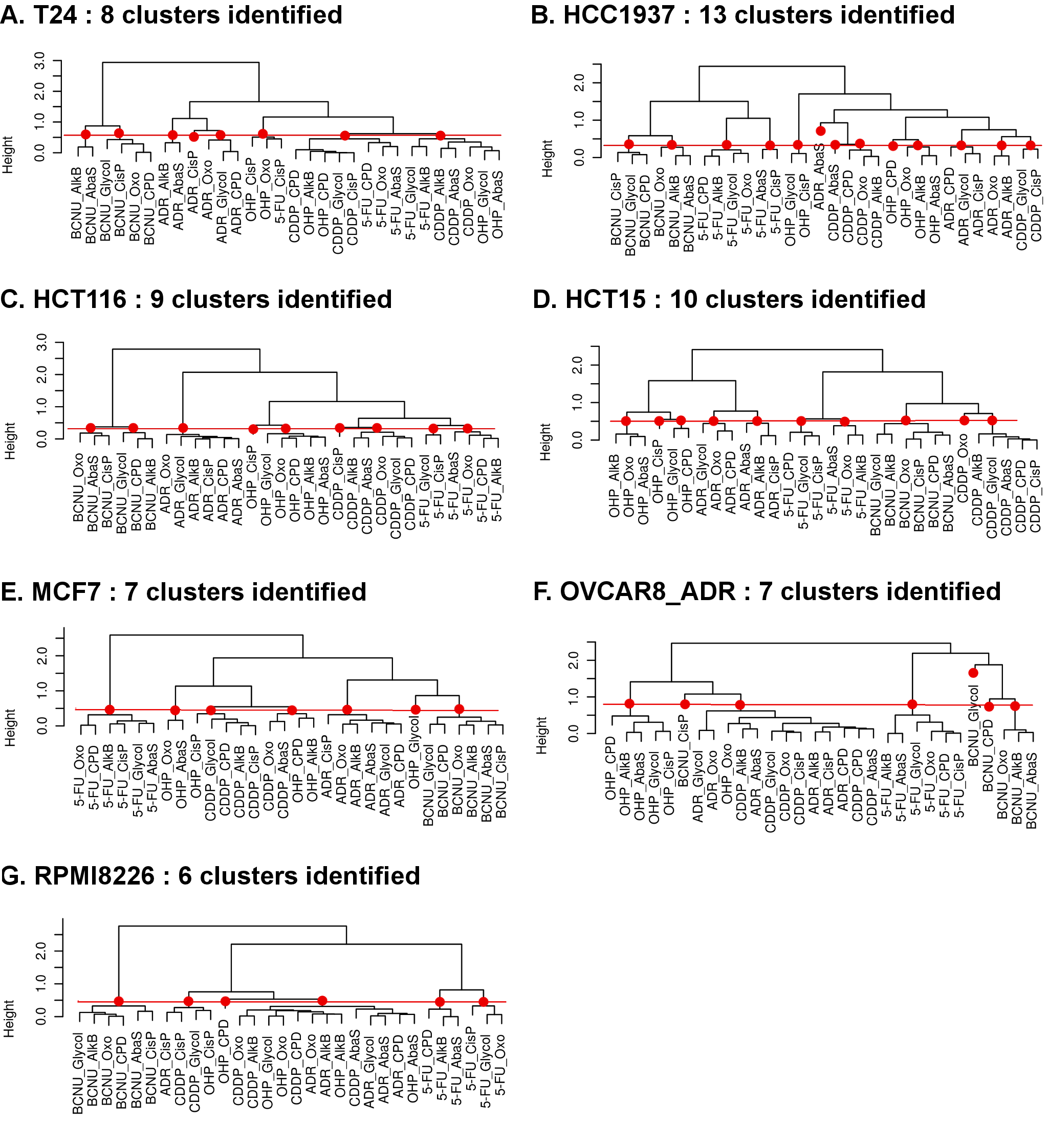

Supplement: Figure S2 — For each cell line, treatments by lesion type were clustered according to their repair response and IC20 (standardized data). Unsupervised hierarchical clustering was performed, using Euclidean dissimilarity measure and average linkage agglomeration method. The cluster dendrogram is displayed together with the agglomeration criteria of average linkage method. To get a partition of the data, the resulting cluster dendrograms were cut at the agglomeration step (represented by the dots of the red line) corresponding to the optimal number of clusters indicated by the agglomeration criteria inflexion point. This latter operation determined the number of clusters identified (A: T24, 8 clusters identified; B: HCC1937, 13 clusters identified; C: HCT-116, 9 clusters identified; D: HCT-15, 10 clusters identified; E: MCF7, 7 clusters identified; F: OVCAR-8/ADR, 7 clusters identified; G: RPMI8226, 6 clusters identified). (TIF) [file pone.0051754.s002.tif]

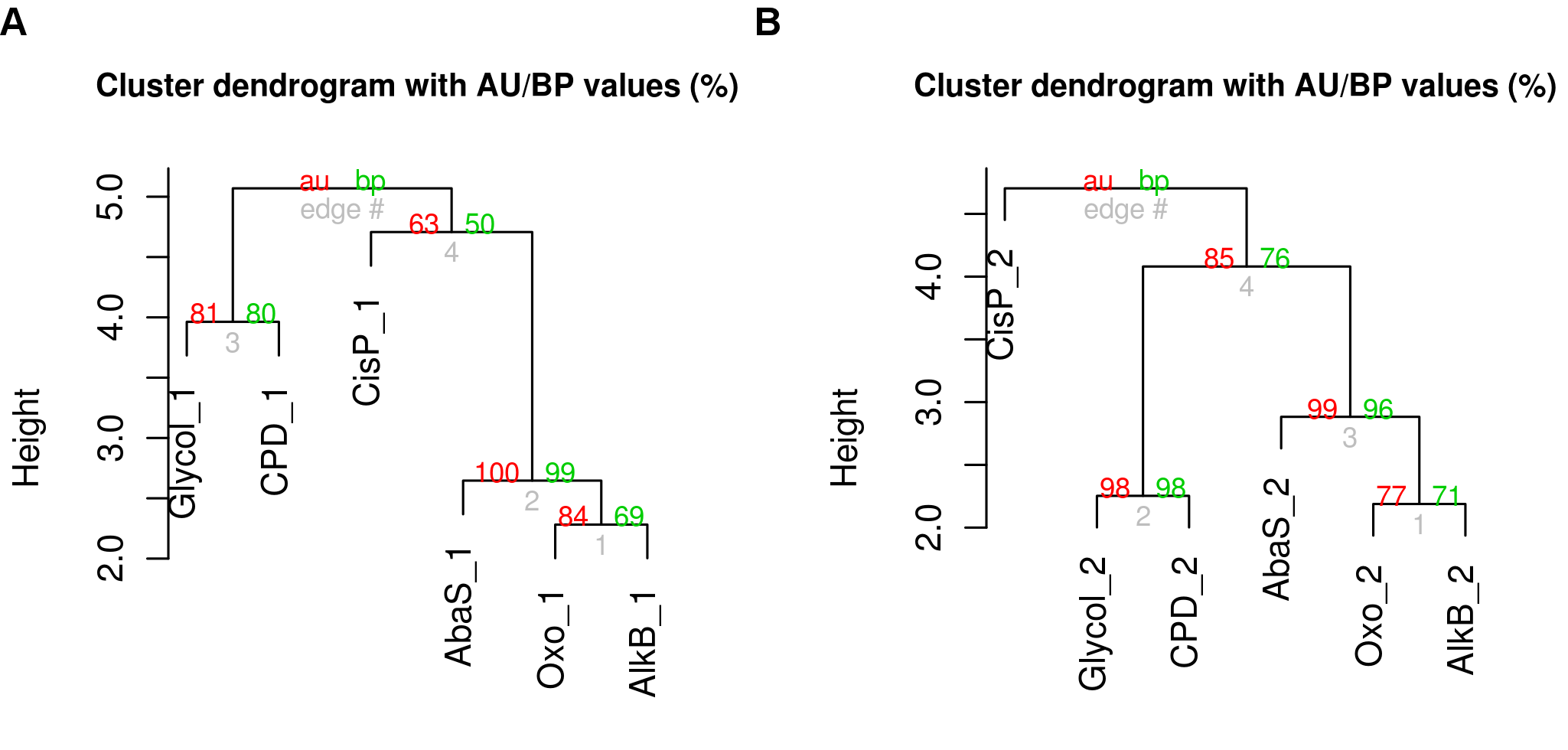

Supplement: Figure S3 — Clustering of the DNA repair pathways, represented by the lesions. The 2 sets of experiments (Set_1 (A) and Set_2 (B), noted _1 and _2) were clustered, using the Euclidian dissimilarity. Similar results were obtained when the correlation dissimilarity was considered. Four treated cell lines presenting unquantifiable repair (very low signals) in Set_2 were removed from the data set since their atypical profiles with very low log2 ratios had an overly strong influence on clustering (RPMI8226_5-FU, HCT-116_ADR, HCT-116_BCNU and MCF7_OHP). (TIF) [file pone.0051754.s003.tif]

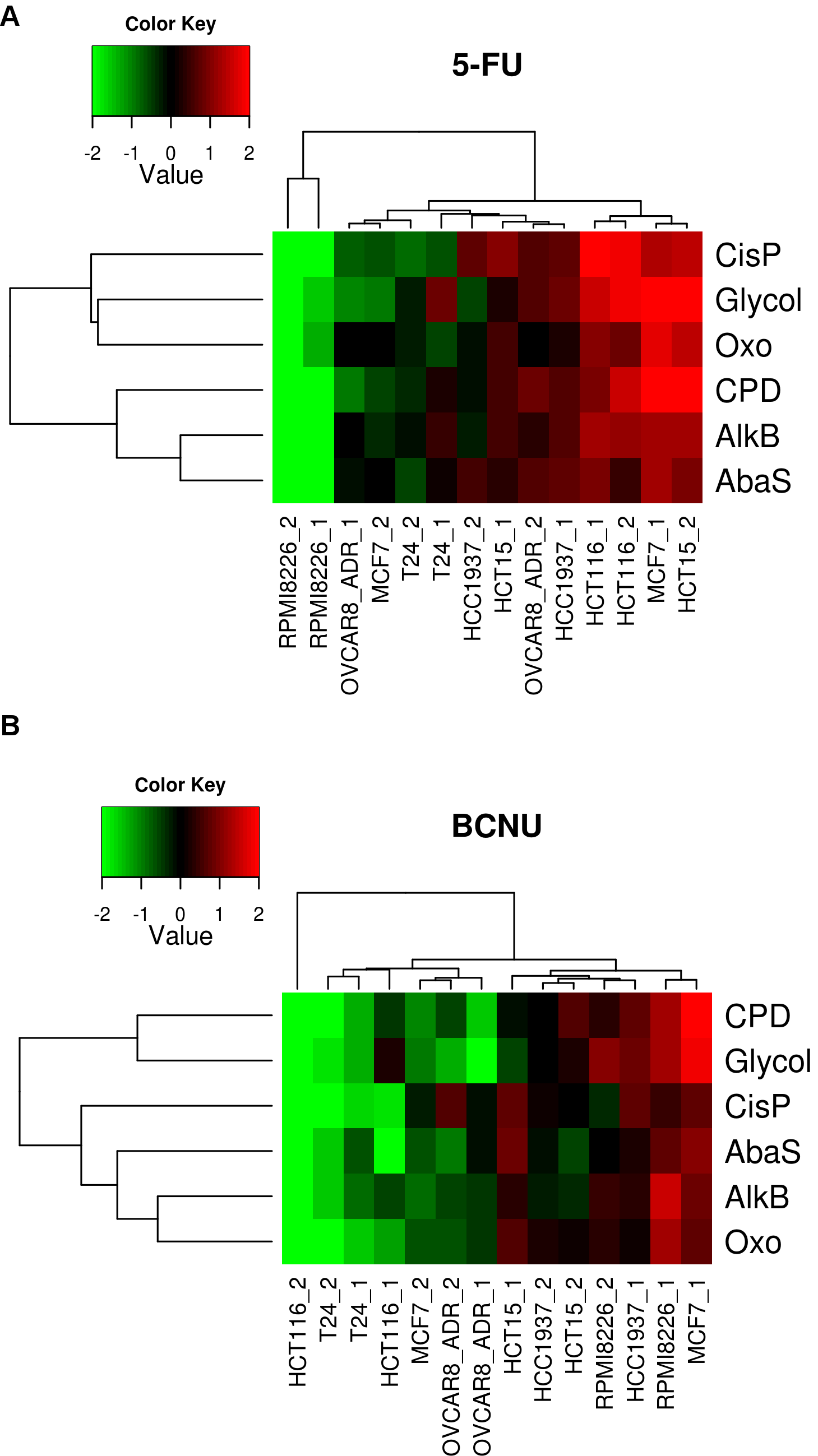

Supplement: Figure S4 — Analysis of the DNA repair response clustering independently for cells treated with 5-FU (A) and BCNU (B) using the Euclidian dissimilarity. This analysis provided additional data for Fig. 3. A. RPMI8226 remained apart, whereas the two other cell line clusters previously identified clustered as a single significant group. The 5-FU treatment, in particular, significantly stimulated all repair activities in the two colon cell lines, HCT-116 and HCT-15 (one-sided Wilcoxon test; P value = 0.0625). B. A new significant cell line cluster sharing similarities in response to BCNU (stimulation of 8oxoG and CPD-64 repair activities) was identified: [HCC1937, RPMI8226, HCT-15] (one-sided Wilcoxon test; P value = 0.05). (TIF) [file pone.0051754.s004.tif]
