# Supplementary material for: Functional DNA Repair Signature of Cancer Cell Lines Exposed to a Set of Cytotoxic Anticancer Drugs Using a Multiplexed Enzymatic Repair Assay on Biochip
Source: PLoS One. 2012 Dec 31;7(12):e51754. doi: 10.1371/journal.pone.0051754 (PMC3534104; doi:10.1371/journal.pone.0051754)
Supplement: Table S1 — Mechanism of action of the drugs used. (DOC) [file pone.0051754.s005.doc]

| **Drug** | **Abbreviation** | **Main reported mechanisms of action and lesions induced** |
| --- | --- | --- |
| Cisplatin | CDDP | Alkylating (intra-strand and inter-strand cross links) |
| Oxaliplatin | OHP | Alkylating (intra-strand and inter-strand cross links) |
| Adryamicin | ADR | Intercalating, free radical generator, affects DNA synthesis. |
|  |  | Topoisomerase II inhibitor |
| 5-Fluorouracil | 5-FU | Antimetabolite, inhibitor of thymidylate synthase, folate. |
|  |  | synthesis inhibitor. Incorporated into DNA and RNA |
| Carmustin | BCNU | Nitrosourea: mono alkylation of guanine and inter-strand |
|  |  | crosslinks |
