# Supplementary material for: Functional DNA Repair Signature of Cancer Cell Lines Exposed to a Set of Cytotoxic Anticancer Drugs Using a Multiplexed Enzymatic Repair Assay on Biochip
Source: PLoS One. 2012 Dec 31;7(12):e51754. doi: 10.1371/journal.pone.0051754 (PMC3534104; doi:10.1371/journal.pone.0051754)
Supplement: Table S2 — IC20 expressed in molar concentration determined by MTS test. (DOC) [file pone.0051754.s006.doc]

|  | **CDDP** | **OHP** | **ADR** | **5-FU** | **BCNU** |
| --- | --- | --- | --- | --- | --- |
| T24 | 1E-06 | 1E-06 | 1E-07 | 2E-06 | 2E-05 |
| HCC1937 | 2E-07 | 7E-09 | 6E-08 | 5E-07 | 2E-05 |
| HCT-116 | 5E-07 | 1E-07 | 7E-08 | 1E-06 | 1E-05 |
| HCT-15 | 2E-06 | 1E-07 | 6E-08 | 5E-07 | 1E-05 |
| MCF-7 | 2E-05 | 7E-06 | 4E-07 | 2E-09 | 5E-05 |
| OVCAR-8/ADR | 4E-06 | 1E-05 | 6E-06 | 2E-09 | 2E-07 |
| RPM8226 | 1E-07 | 7E-08 | 8E-08 | 1E-07 | 1E-05 |

**Supplementary Figures**

**Figure S1. Experimental workflow**

**Figure S2.** For each cell line, treatments by lesion type were clustered according to their repair response and IC20 (standardized data). Unsupervised hierarchical clustering was performed, using Euclidean dissimilarity measure and average linkage agglomeration method. The cluster dendrogram (left panel) and agglomeration criteria of average linkage method (right panel) are displayed. To get a partition of the data, the resulting cluster dendrograms were cut at the agglomeration step corresponding to the optimal number of clusters indicated by the agglomeration criteria inflexion point. This latter operation determined the number of clusters identified (A: T24; B: HCC1937; C: HCT-116; D: HCT-15; E: MCF7; F: OVCAR-8/ADR; G: RPMI8226).

**A. T24: 8 clusters identified**


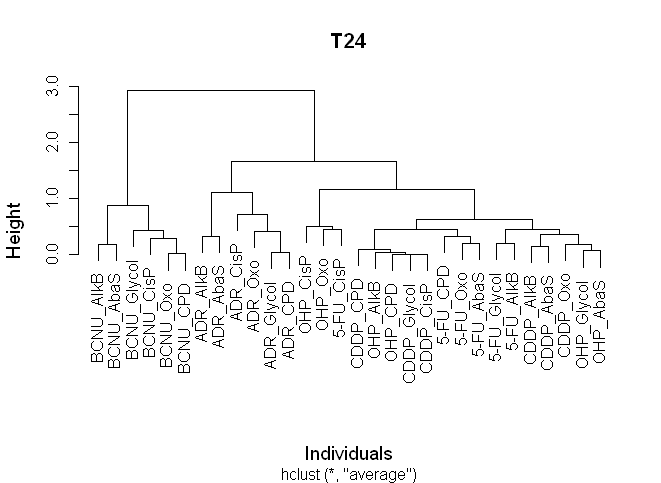

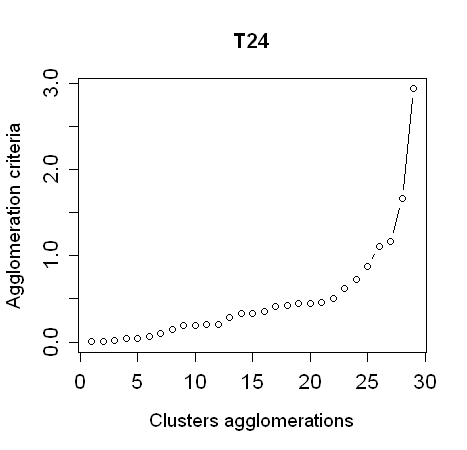


**B. HCC1937: 13 clusters identified**


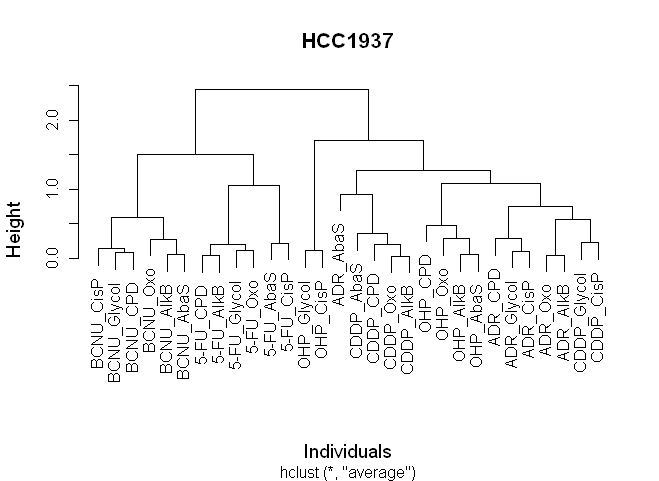

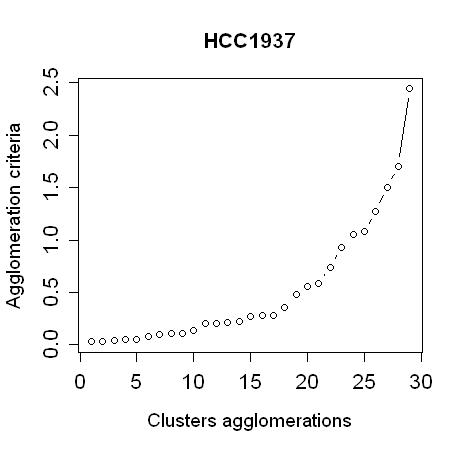


**C. HCT-116: 9 clusters identified**


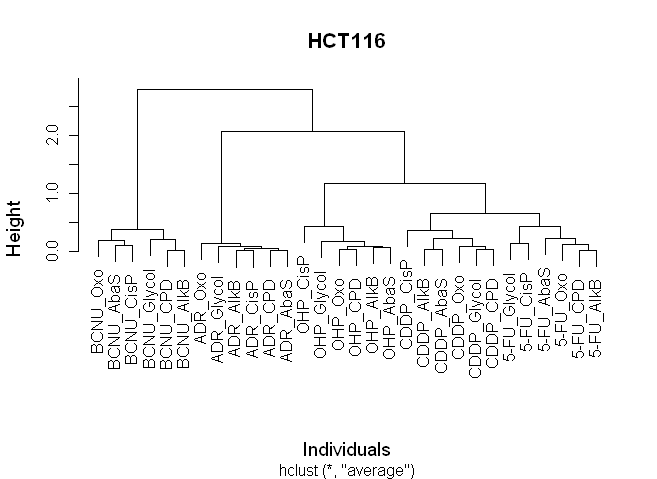

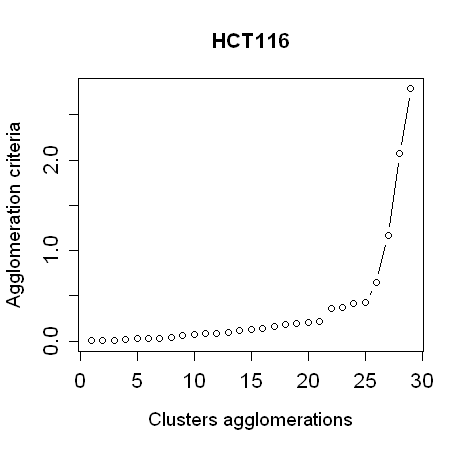


**D. HCT-15: 10 clusters identified**


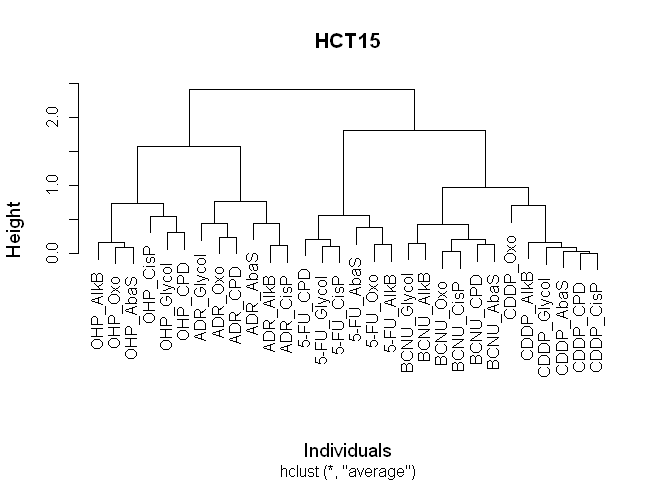

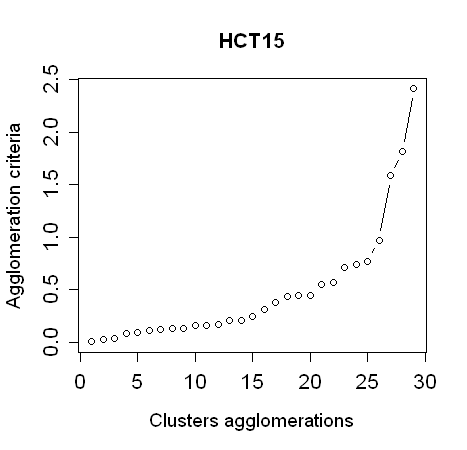


**E. MCF7: 7 clusters identified**


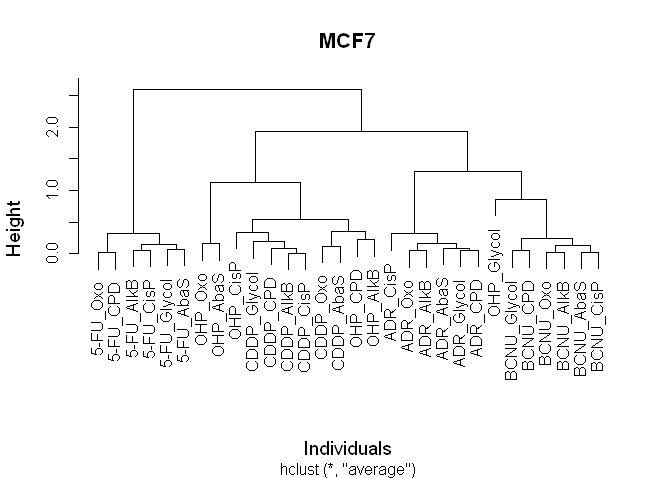

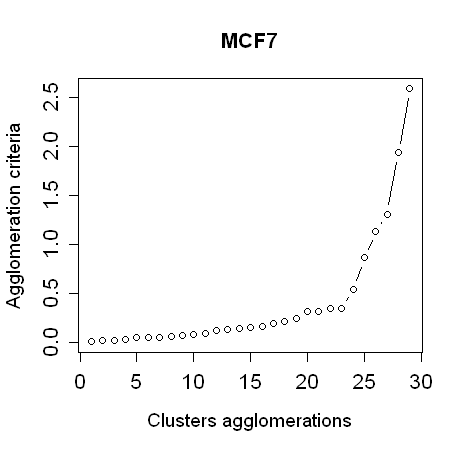


**F.** OVCAR-8/ADR**: 7 clusters identified**


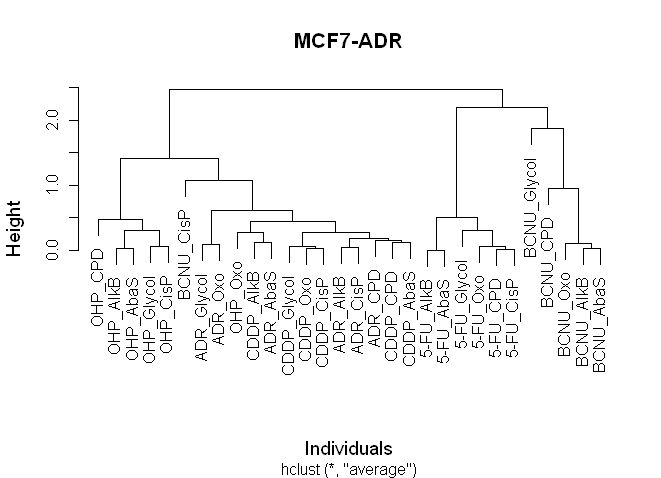

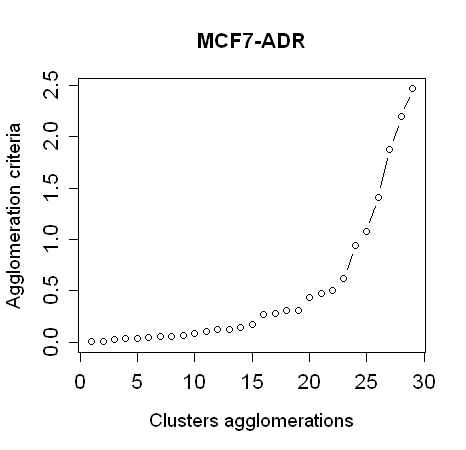


**G. RPMI8226: 6 classes identified**


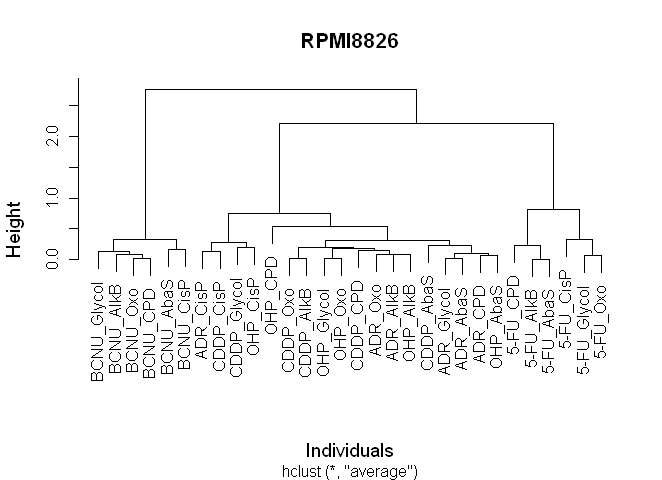

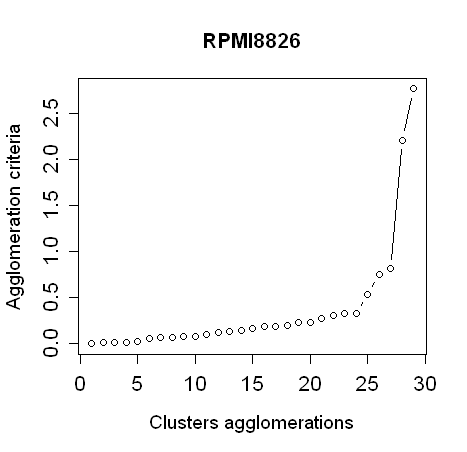


**Figure S3.** Clustering of the DNA repair pathways, represented by the lesions, for the 2 sets of experiments (Set_1 (A) and Set_2 (B), noted _1 and _2), using the Euclidian dissimilarity. Similar results were obtained when the correlation dissimilarity was considered. Four treated cell lines presenting unquantifiable repair (very low signals) in Set_2 were removed from the data set since their atypical profiles with very low log2 ratios had a too strong influence on clustering (RPMI8226_5-FU, HCT-116_ADR, HCT-116_BCNU and MCF7_OHP).

**A**

**B**

| 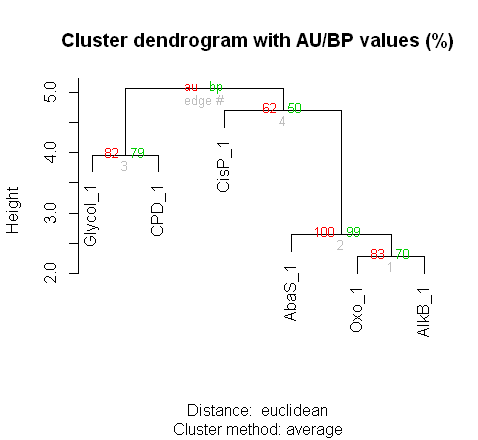 | 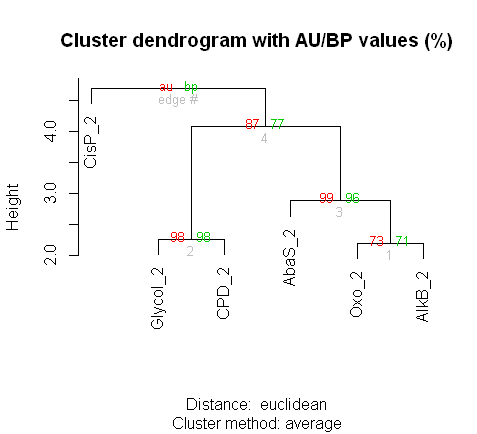 |
| --- | --- |

**Figure S4.** Cluster analysis of the DNA repair response independently for 5-FU (A) and BCNU (B) treated cells using the Euclidian dissimilarity. This analysis brought additional data to Fig. 3. A. RPMI8226 remained apart whereas the two other cell line clusters previously identified were joined in a single significant group. We observed in particular that 5-FU treatment induced a significant stimulation of all repair activities of the two colon cell lines HCT-116 and HCT-15 (one-sided Wilcoxon test; *P* value = 0.0625). B. A new significant cell line cluster sharing similarities in response to BCNU (stimulation of 8oxoG and CPD-64 repair activities) was identified: [HCC1937, RPMI8226, HCT-15] (one-sided Wilcoxon test; *P* value = 0.05).

| **A**  **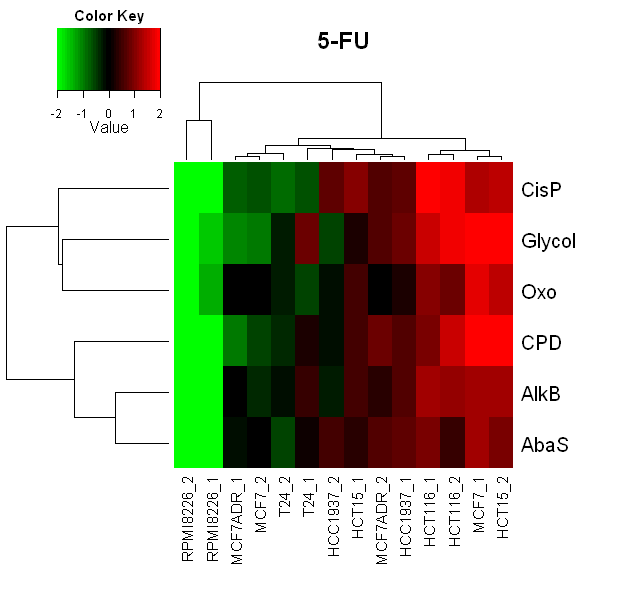** |
| --- |
| **B** |
| **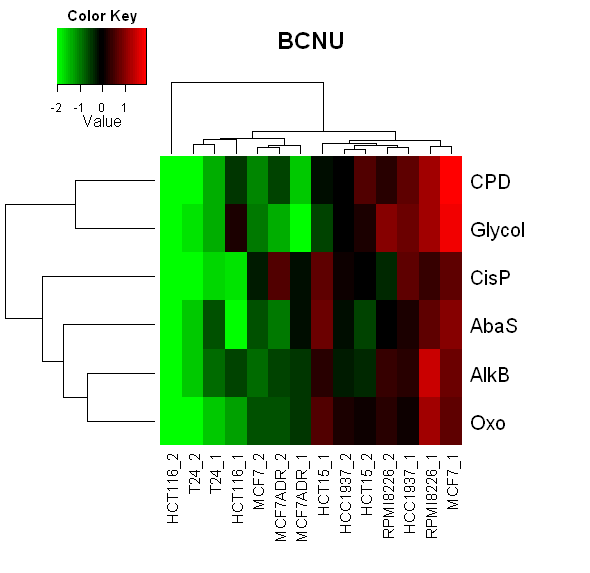** |
