# Supplementary material for: Functional DNA Repair Signature of Cancer Cell Lines Exposed to a Set of Cytotoxic Anticancer Drugs Using a Multiplexed Enzymatic Repair Assay on Biochip
Source: PLoS One. 2012 Dec 31;7(12):e51754. doi: 10.1371/journal.pone.0051754 (PMC3534104; doi:10.1371/journal.pone.0051754)
Supplement: Methods S1 — Supplementary information on the following: Chemosensitivity assay, modified plasmid microarray, preparation of cell nuclear extracts, DNA excision/synthesis reaction, data normalization, statistical tests to evaluate degree of similarity, data analysis - clustering methods - results display. (DOC) [file pone.0051754.s007.doc]

**Supporting Information**

**Supplementary Methods**

***Chemosensitivity assay.*** Cells were plated (5 000 to 10 000 cells per well according to doubling time) in microtitration plates (Nunc, France) and incubated in a 5% CO_2_ atmosphere at 37°C for 24 h in 200 µL of RPMI-1640 medium. Ten cascade dilutions of test substances were prepared in the culture medium and the cells were further incubated for 72 h in the CO_2_ incubator (5%). At the end of the treatment 40 µL of MTS/PMS solution (at 2 mg/mL and 0.5 mg/mL respectively in PBS) were added in each well further incubated 2h at 37°C. Each concentration was tested in quadruplicate (Oncodesign, France).

***Modified plasmid microarray.*** Each microarray comprises series of lesion-containing plasmids and a non modified control plasmid (CTRL). Six lesions were generated on different plasmids before spotting: photoproducts (cyclobutane pyrimidine dimers and (6-4)photoproducts; CPD-64 plasmid), 8-oxoguanine (8oxoG plasmid), alkylated bases (AlkB plasmid), cisplatin adducts (CisP plasmid), abasic sites (AP sites; AbaS plasmid) and cytosine and thymine glycols (Glycol plasmid). Each type of modified plasmid was then diluted in non-modified plasmid so we obtained 3 solutions of identical DNA concentration (40 µg/mL) but with 3 different ratios lesion/DNA (ratios ½, ¾ and 1, called A, B and C dilutions, respectively). On all microarrays each plasmid solution was spotted duplicated except the CTRL that was deposited 9 times.

***Preparation of cell nuclear extracts.*** Cell nuclear extracts were prepared as already described [32,36]. Thawed cells were washed twice in ice-cold PBS and incubated on ice for 20 min in 1.25 mL of ice-cold buffer A (10 mM HEPES pH 7.9, 1.5 mM MgCl_2_, 10 mM KCl, 0.01% Triton X-100, 0.5 mM DTT, 0.5 mM PMSF). The cytosolic membrane was disrupted by vortexing the tube for 30 s. Nuclei were recovered by centrifugation for 5 min at 5000 rpm and 4 °C. The pellet was suspended in 31.25 µL of ice-cold buffer B (10 mM HEPES pH 7.9, 1.5 mM MgCl_2_, 400 mM KCl, 0.2 mM EDTA, 25% glycerol, 0.5 mM DTT, antiproteases (Complete-mini, Roche, France) and 0.5 mM PMSF). Nuclear membranes were lysed for 20 min on ice, and two cycles of freezing-thawing at -80 °C and 4 °C respectively. The extracts were cleared by centrifugation for 10 min at 13000 rpm and 4 °C. The cleared supernatant was recovered and stored frozen in 10 µl aliquots at -80 °C. Protein content was determined using the BCA kit (Interchim, France). Typical protein content was 0.8 mg/mL.

***DNA excision/synthesis reaction.*** Custom reaction chambers (Grace Bio-Labs, USA) were set on the damaged plasmid microarray slides and filled with 20 µL of the excision/synthesis mix composed of reaction buffer (5 µL of 5X ATG buffer [200 mM Hepes KOH pH 7.8, 35 mM MgCl_2_, 2.5 mM DTT, 1.25 µM dATP, 1.25 µM dTTP, 1.25 µM dGTP, 17% glycerol, 50 mM phosphocreatine (Sigma, USA), 10 mM EDTA, 250 µg/mL creatine phosphokinase, 0.5 mg/mL BSA]), 1 mM ATP (Amersham, England), 1.25 µM dCTP-Cy5 (Amersham, England) and containing the nuclear extract at a final protein concentration of 0.2 mg/mL. The slides were incubated for 3 h at 30 °C in the dark. The reaction chambers were then removed and the slides were washed for 2 x 3 min in PBS/Tween 0.05%, and for 2 x 3 min in MilliQ water. Slides were then centrifuged 3 min at 700 rpm, and allowed to dry 5 min at 30 °C.

***Data normalization*** (See Experimental Workflow in this Supporting Information section Fig. S1). A first normalization was performed on replicate HeLa_Com data obtained each day, using the NormalizeIt software specifically developed for this purpose [36]. Thus, one set of normalized HeLa_Com data were obtained per day. Eventually, each set of normalized data comprised one control and three dilutions per modified plasmid value i.e., 19 values. To limit the scale variation between data points, the FI was log 10 transformed. The three series obtained within each independent set of repair reactions (Set_1 and Set_2) were then compared. For this purpose, one series of data was taken as reference and a linear regression was used to model the bias between the reference series and each of the other series. The resulting linear coefficients, when significantly different from 0, were then applied to adjust each series to the reference one. No significant difference between the 3 series of Set_2 and between series 1 and 2 of Set_1 were found. They were thus normalized within each Set using NormalizeIt. In addition, HeLa_Com data from Set_1 was further normalized taking the normalized data from series 1 and 2 as reference and applying the coefficients calculated from the linear regression to correct series 3 of Set_1. A similar approach was then used to calculate the correcting coefficients between the reference data (normalized HeLa_Com series 1 and 2 of Set_1) and the HeLa_Com dataset from Set_2.

Each set of normalized data collected from the cancer cell line experiments were log-transformed (base 10) and the datasets belonging to Set_2 and to series 3 of Set_1 were subsequently corrected according to the correction coefficients calculated with HeLa_Com data.

***Statistical tests to evaluate degree of similarity.*** We used the "pvclust" R package to assess the uncertainty in hierarchical cluster analysis (http://www.is.titech.ac.jp/~shimo/prog/pvclust/). For each cluster, this package calculates p-values using a bootstrap resampling method, indicating how strongly the cluster is supported by data. Two types of *P* values are provided: BP (Bootstrap Probability) and AU (Approximately Unbiased) *P* values. The AU *P* value, which is computed by multiscale bootstrap resampling, is a better approximation of the unbiased *P* value than the BP value, which is computed by normal bootstrap resampling. They are displayed (%) on the edges of the clustering (red: AU *P* values; green: BP values). For a cluster with AU *P* value > 0.95, the hypothesis that "the cluster does not exist" is rejected with significance level of 0.05, i.e. this cluster is strongly supported by data.

***Data analysis - Clustering methods - Results display.*** The hierarchical average linkage clustering algorithm uses an iterative process clustering items by profile similarity. The results are displayed in a dendrogram format. Items with similar profiles are placed in adjacent dendrogram nodes, connected by branches proportional in length to the corresponding dissimilarity measure. A data table is usually clustered along its two dimensions, in order to complete profile clustering by clustering profile variables. The resulting clustered data table is displayed as a heatmap with the two dendrograms along its two dimensions. This allows cluster trees and cluster profiles to be viewed simultaneously. In heatmap format, data values are colour-coded, from green (low data) to red (high data), the colour brightness correlates with the data point's value.
