# Supplementary material for: Functional DNA Repair Signature of Cancer Cell Lines Exposed to a Set of Cytotoxic Anticancer Drugs Using a Multiplexed Enzymatic Repair Assay on Biochip
Source: PLoS One. 2012 Dec 31;7(12):e51754. doi: 10.1371/journal.pone.0051754 (PMC3534104; doi:10.1371/journal.pone.0051754)
Supplement: Supplementary file 5 [file pone.0051754.s001.doc]

Repair Assay Set_1

7 cell lines x (5 T + 1 NT)
48h treatment (IC20)

↓

42 Cell Pellets _1
42 Cell Nuclear Extracts _1

↓

Repair Assay_1

84 Cell Extracts to be tested
(replicates = 42x2)

3 days testing, 4 slides/ day

D1_1 D2_1 D3_1

12 samples /slide { 3 Standard Extracts
9 Cell Extracts }

Fluorescence quantification

Normalization (NormalizeIt) of the replicates

Repair Assay Set_2

7 cell lines x (5 T + 1 NT)
48h treatment (IC20)

↓

42 Cell Pellets _2
42 Cell Nuclear Extracts _2

↓

Repair Assay_2

84 Cell Extracts to be tested
(replicates = 42x2)

3 days testing, 4 slides/ day

D1_2 D2_2 D3_2

12 samples /slide { 3 Standard Extracts
9 Cell Extracts }

Fluorescence quantification

Normalization (NormalizeIt) of the replicates

Inter-slide and inter-experiment normalization

Normalization (NormalizeIt) of HeLa_Com data per day

Log10 transformation of Standard Extract (HeLa_Com) data

HeLa_Com_1_1 HeLa_Com_2_1 HeLa_Com_3_1 HeLa_Com_1_2 HeLa_Com_2_2 HeLa_Com_3_2

Calculation of linear model coefficients to correct HeLa_Com dataset

Log10 transformation of Cell Extract data

Application of the coefficients to adjust the Cell Extract dataset

Log10 back-transformation of Cell Extract data

Data analysis

Calculation of the fluorescence Intensity (FI) for repair of each lesion
(sum of the 3 corresponding plasmid dilutions)

Subtraction of 3*CTRL Plasmid FI

Calculation of the ratio T/NT for repair of each lesion

Log2 transformation of T/NT

Clustering methods
